# Considerations and a call to action for the use of noncontact forehead infrared handheld thermometers during the COVID-19 pandemic

**DOI:** 10.7189/jogh.11.03023

**Published:** 2021-01-30

**Authors:** Wing P Chan, Russell Oliver Kosik, C Jason Wang

**Affiliations:** 1 Department of Radiology, Wan Fang Hospital, Taipei Medical University, Taipei, Taiwan; 2 Department of Radiology, School of Medicine, College of Medicine, Taipei Medical University, Taipei, Taiwan; 3 Departments of Pediatrics, Medicine, and Health Research and Policy, Stanford University School of Medicine, Stanford, California, USA; 4 The New School for Leadership in Health Care, Koo Foundation Sun Yat-Sen Cancer Center, Taipei, Taiwan

Early in January 2020, the WHO announced that temperature screening at entry was sufficient to detect the majority of imported COVID-19 cases [1]. As such, several countries began employing thermal screening in airports and public places as a preventative measure against the spread of coronavirus. A February 20th report from the WHO noted that among 55 924 laboratory confirmed cases of COVID-19 in China, 87.9% had a “fever” (no cutoff point reported), and people with COVID-19 generally developed signs (including fever) and symptoms on average 5-6 days following exposure (range 1-14 days or even longer). However, there is a lack of a universally accepted definition of what exactly constitutes “fever” when a noncontact infrared thermometer is used.

Prior research has shown that a forehead temperature in excess of 35.6 [2], or 36.2°C if measured indoors [3], is suggestive of fever. The definition of fever remains unclear however, as body temperature is influenced by many factors including site and time of measurement, one’s environment (indoors vs outdoors), activities performed prior to measurement, age, ovulatory cycle, type of thermometer used, and expected variations between and within individuals. For example, older adults’ (age ≥60) temperatures are 0.23°C lower than younger adults (age <60) on average [4]. Most authors [5,6] agree that the mean normal oral temperature is 36.8 ± 0.4°C (98.2°F). If this threshold is breached (early morning: >37.2°C or >99°F; any other time during the day: 37.7°C or >100°F), which is roughly equivalent to a rectal temperature of ≥38°C (100.4°F) and an axillary temperature of ≥37.5°C (99.5°F), a fever is present as defined by the WHO [7]. Of the three major measuring sites (ie, rectal, oral, and axillary), rectal temperatures most accurately estimate core temperature [6]. Yet, there are no guidelines to allow for comparison of body temperatures across different body sites, including the forehead.

Additionally, even though handheld noncontact infrared thermometers are convenient and safe, they are operator dependent and offer low sensitivity (29.4%) compared to oral temperature measurements [8]. Some manufacturers have suggested an optimum measurement distance of 2 to 6 inches or 5 to 15 cm when placed perpendicular to the forehead. Ng et al. [2] measured the forehead temperatures of 1000 healthy individuals using handheld infrared thermometers and determined that 35.6°C should serve as a cutoff measurement between healthy and febrile individuals. Chen et al. [3] recorded the tympanic temperatures of 528 (261 indoor and 267 outdoor) participants to be used as a standard for forehead infrared measurements. When a tympanic temperature of 37.3°C or above was considered febrile, a corresponding cut-off value of 36.2°C when using a contact infrared thermometer at the wrist was determined (86.4% sensitivity and 67.0% specificity). Similarly, this corresponded to a cut-off value of 36.2°C when measured at the forehead indoors (93.2% sensitivity and 60.0% specificity) [3]. This work implies that reference ranges for fever measured with infrared technology can vary substantially by body site.

**Figure:**
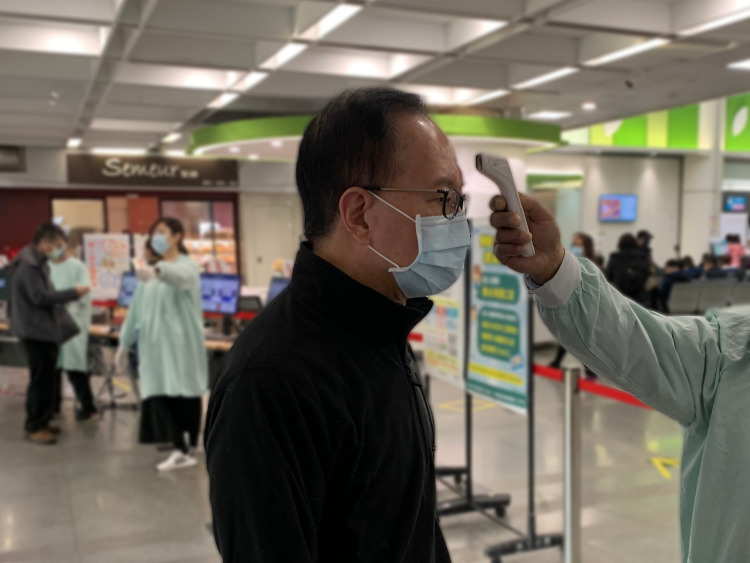
Photo: Temperature screening at the hospital entrance (image by Wing Chan, used with permission).

Ideally, equivocal temperatures identified during screening in public places using handheld infrared thermometers should be rechecked with a tympanic thermometer or an oral thermometer. Further, adequate reference information for infrared screening would reduce the rate of false negatives.

Forehead measurements with infrared technology offer the convenience of contactless mass screening and do not require subjects to remove their clothes. However, validation of the accuracy and precision of this technology across different instruments, body sites, and environments is urgently needed. Moreover, the threshold for fever using infrared technology may be lower than those used for tympanic and oral measurements. In cities all over the world, tens of thousands of temperature measurements are performed daily. If just a fraction of this data were recorded and made available to investigators, it would provide invaluable information concerning the range of normal body temperature. Further, because many people are measured multiple times per day and in different settings, such data could offer insights into how temperatures vary over time and across settings. This data would allow those in the public health space to select more appropriate cutoff temperatures in screening for potential COVID-19 infections. While collecting and organizing such data would certainly require some effort, the pandemic offers a unique opportunity to do so, and it should not be wasted.
